# Replacement of carbohydrate binding modules improves acetyl xylan esterase activity and its synergistic hydrolysis of different substrates with xylanase

**DOI:** 10.1186/s12896-016-0305-6

**Published:** 2016-10-22

**Authors:** Shiping Liu, Shaojun Ding

**Affiliations:** College of Chemical Engineering, Nanjing Forestry University, Nanjing, 210037 Jiangsu China

**Keywords:** Xylan, Acetyl xylan esterase, Carbohydrate-binding module, Xylan-specific, Fusion enzyme, Synergism

## Abstract

**Background:**

Acetylation of the xylan backbone was a major obstacle to enzymatic decomposition. Removal of acetyl groups by acetyl xylan esterases (AXEs) is essential for completely enzymatic hydrolysis of xylan. Appended carbohydrate binding modules (CBMs) can promote the enzymatic deconstruction of plant cell walls by targeting and proximity effects. Fungal acetyl xylan esterases are strictly appended to cellulose-specific CBM1. It is still unclear whether xylan-specific CBMs have a greater advantage than CBM1 in potentiating the activity of fungal deacetylating enzymes and its synergistic hydrolysis of different substrates with xylanase.

**Results:**

Three recombinant AXE1s fused with different xylan-specific CBMs, together with wild-type AXE1 with CBM1 and CBM1-deleted mutant AXE1dC, were constructed in this study. The optimal temperature and pH of recombinant AXE1s was 50 °C and 8.0 (except AXE1dC-CBM6), respectively. Cellulose-specific CBM1 in AXE1 obviously contributed to its catalytic action against substrates compared with AXE1dC. However, replacement of CBM1 with xylan-specific CBM4-2 significantly enhanced AXE1 thermostability and catalytic activity against soluble substrate 4-methylumbelliferyl acetate. Whereas replacements with xylan-specific CBM6 and CBM22-2 were more effective in enzymatic release of acetic acid from destarched wheat bran, NaClO_2_-treated wheat straw, and water-insoluble wheat arabinoxylan compared to AXE1. Moreover, replacement with CBM6 and CBM22-2 also resulted in higher degree releases of reducing sugar and acetic acid from different substrates when simultaneous hydrolysis with xylanase. A good linear relationship exists between the acetic acid and reducing sugar release.

**Conclusions:**

Our findings suggested that the replacement with CBM6 and CBM22-2 not only significantly improved the catalysis efficiency of AXE1, but also increased its synergistic hydrolysis of different substrates with xylanase, indicating the significance of targeting effect in AXE1 catalysis mediated by xylan-specific CBMs.

**Electronic supplementary material:**

The online version of this article (doi:10.1186/s12896-016-0305-6) contains supplementary material, which is available to authorized users.

## Background

Xylan is the major constituent of hemicellulose and the second most abundant renewable resource in nature. Xylan serves as the source of C-5 sugars used in the production of biofuels, xylooligosaccharides or other chemicals [1]. Xylan generally contains heterogeneous substituents such as L-arabinose, O-acetyl, ferulic (4-hydroxy-3- methoxycinnamic) acid, *p*-coumaric (4-hydroxycinnamic) acid and 4-O-methyl-D-glucuronic acid [2, 3]. Therefore, complete hydrolysis of xylans requires not only glycoside hydrolases (endo-β-1,4-xylanases, β-xylosidases), but also deacetylating enzymes, namely, acetyl xylan esterases (EC 3.1.1.72, AXEs), and other side substituent cleaving enzymes, including α-L-arabinofuranosidases (EC 3.2.1.55), α-glucuronidases (EC 3.2.1.139) and ferulic/coumaric acid esterases (EC 3.1.1.73) [4]. Acetylation of the xylan backbone was thought to be a major obstacle to enzymatic decomposition [5, 6]. The role of acetyl xylan esterases (AXEs) is to remove acetyl groups and create new sites for productive binding of glycoside hydrolases, consequently enhancing xylan accessibility and enabling complete hydrolysis [7].

Synergistic actions between xylanases and acetyl xylan esterases have been reported. An acetyl xylan esterase (BnaA) from *Neocallimastix patriciarum* showed a significant synergistic effect in combination with a recombinant xylanase (XynA) on the degradation of delignined spear grass. More reducing sugars were released by adding BnaA and XynA simultaneously than by XynA alone [8]. Tong et al. [9] found that the overall xylose yield from wheat arabinoxylan hydrolysis was 8 % with xylanase treatment and increased to 34 % when xylanase was combined with AXE from *Chaetomium thermophilum*. Furthermore, such enzymatic release of acetyl groups by AXE not only enhanced the solubilization of xylan to some extent and, but also increased the subsequent hydrolysis of cellulose by cellulases [10].

In general, cellulases and hemicellulases are modular enzymes in which the catalytic domain is appended to one or more noncatalytic carbohydrate binding modules (CBMs) [11]. CBMs have the ability to enhance the hydrolysis of insoluble carbohydrate by concentrating the parental enzyme at their target polysaccharide [12, 13]. The Carbohydrate Active Enzyme (CAZy) database (http://www.cazy.org/Carbohydrate-Binding-Modules) currently lists 74 CBM families classified based on amino acid similarity [14]. Binding specificity can vary both between and within families [15, 16], for example, CBM1 binds specifically to cellulose [17], whereas CBM 4-2, 6 and 22-2 bind specifically to xylan [18–20].

The bacterial cellulolytic and hemicellulolytic enzymes contain various CBMs with diverse polysaccharide recognition specificity, paralleling the considerable diversity in the target substrate of the catalytic module of the enzymes. Thus, the catalytic domains from cellulases [21, 22], xylanases [23] and mannanases [24] are generally appended to cellulose, xylan and mannan-binding CBMs, respectively. Therefore, different types of CBMs can assist the appended catalytic domain targeting to a particular substrate and potentiate the catalytic rates of enzymes [25–29]. In contrast, the catalytic domains in fungal cellulolytic and hemicellulolytic enzymes are strictly appended to CBM1. That is, numerous enzymes that do not cleave cellulose contain CBM1 that recognize the celluolose [30, 31]. CBM1 can potentiate enzyme activity of the catalytic module against hemicellulose due to its proximity effects, because in intact plant cell wall, hemicelluloses are in close association with cellulose [32].

However, this potentiation by targeting and proximity effect of CBMs might rely on the types of enzymes, and probably substrates as well [33, 34]. For example, CBM15, a xylan-binding module, can potentiate the activity of Xyl11A from *Neocallimastix patricarium* but not that of Xyl10B from *Cellvibrio mixtus* [32]. The enhancements of enzyme activity by additional CBM were extensively studied in recent years, but most of them were related to the main-chain degrading enzymes such as cellulases and xylanases [35–38]. It is still unclear whether xylan-specific CBMs are more effective than CBM1 in potentiating the activity of deacetylating enzymes by targeting and proximity effects when against substrates with different structures and cellulose contents. With this end, an acetyl xylan esterase (AXE1) from *Volvariella volvacea*, which consisted of a family 1 catalytic domain and a CBM1 linked with serine and threonine-rich peptide, was investigated in this study [39]. Three CBMs from family 4, 6, and 22 [40–42], respectively, which display different specificity for xylans, were selected to replace CBM1 in AXE1. The influences of CBMs replacement on its enzyme activity and its synergistic hydrolysis of different substrates with xylanase were comparatively studied. The replacement of CBM1 in AXE1 with xylan-specific binding modules resulted in a better catalysis performance than wild-type enzyme did, which provided a rational method to design better AXEs via engineered fusion to CBMs with different binding specificities.

## Results and discussion

### Recombinant enzymes

**Fig. 1:**
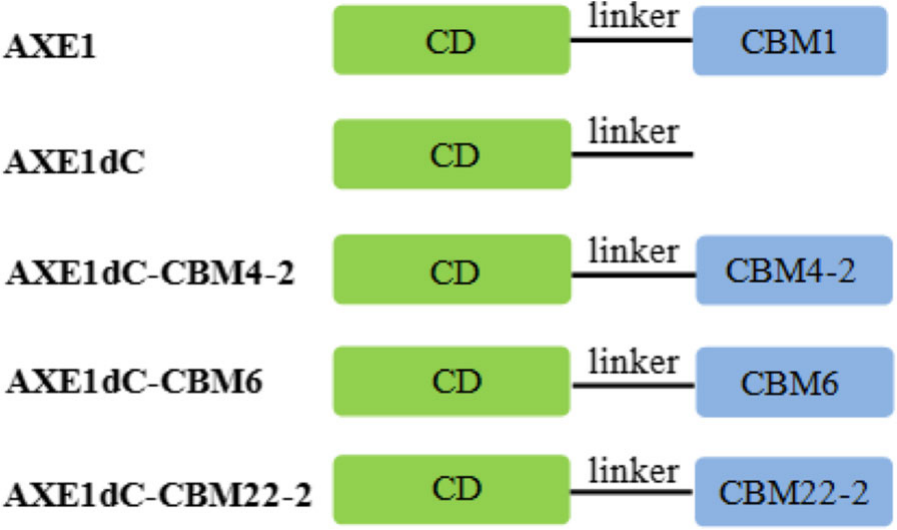
Schematic structures of five recombinant acetyl xylan esterases with and without CBMs. *CD* catalytic domain; linker, linker peptide, *CBM1/4-2/6/22-2* family 1/4-2/6/22-2 carbohydrate-binding modules

To evaluate the influences of CBMs replacement on enzyme activity and its synergistic hydrolysis for different substrates with xylanase, five recombinant AXE1s with and without CBMs were designed, that is, AXE1, a wild-type enzyme; AXE1dC, a CBM1-deleted form but containing the linker; and AXE1dC-CBM4-2/6/22-2, a fusion of AXE1dC and CBM4-2/6/22-2, respectively. Schematic representations of five rAXE1s were shown in Fig. 1.

### Purification and molecular weight analysis

**Fig. 2:**
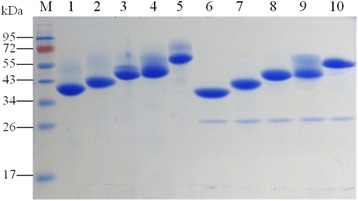
SDS-PAGE of purified rAXE1s and endo H-treated rAXE1s. The gel was stained with Coomassie Brilliant Blue and the samples were loaded in the following order (from left to right): *M*, protein markers, *Lane 1–5*, purified AXE1dC, AXE1, AXE1dC-CBM6, AXE1dC-CBM4-2, AXE1dC-CBM22-2; *Lane 6–10*, endoglycosidase-H-treated rAXE1s, and the bright bands near 26 kDa were endo H

The recombinant proteins were purified in a one-step procedure by affinity chromatography using Ni-NTA agarose gel. The theoretical molecular weights for purified rAXE1s were estimated to be 32.3 kDa (AXE1dC), 36.4 kDa (AXE1), 46.5 kDa (AXE1dC-CBM6), 50.5 kDa (AXE1dC-CBM4-2) and 50.6 kDa (AXE1dC-CBM22-2), respectively. However, SDS-PAGE analysis revealed that the purified enzymes migrated as two bands. The two forms were attributed to different degrees of glycosylation at the two N-glycosylation sites present in the protein. After endoglycosidase H (endo H) treatment, the proteins became a single band except AXE1dC-CBM4-2. But the molecular masses of rAXE1s were still slightly higher than the estimated ones (Fig. 2). The incomplete deglycosylation of rAXE1s may be due to O-glycosylation sites located on rAXE1s, which were found by *O*-β-GlcNAc attachment sites during the eukaryotic protein sequence determination using the YinOYang 1.2 tool (http://www.cbs.dtu.dk/services/YinOYang/).

### Specific activity and biochemical properties of rAXE1s towards soluble substrate

**Fig. 3:**
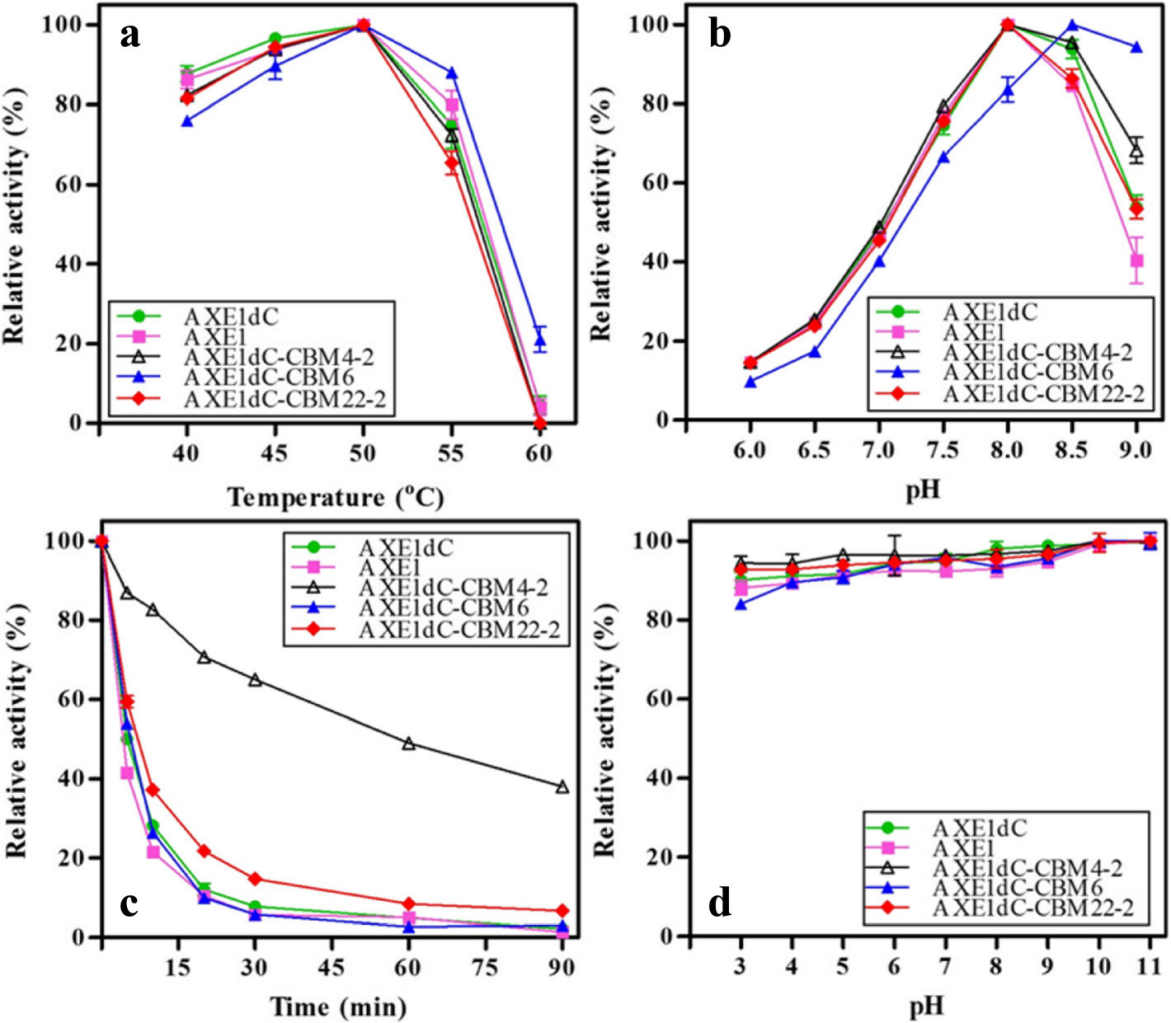
Biochemical properties of rAXE1s. **a** Optimum temperature; **b** Optimum pH; **c** Thermostability; **d** pH stability

The biochemical properties of rAXE1s were compared by using 4-methylumbelliferyl acetate as the substrate. Temperature optima for all rAXE1s were 50 °C and pH optima for rAXE1s were pH 8.0 except AXE1dC-CBM6, which was pH 8.5 (Fig. 3a and b). AXEdC-CBM4-2 retained almost 40 % of activity after incubation at 55 °C for 90 min, in contrast, AXE1 retained only 3 %, indicating that replacement of CBM1 with CBM4-2 resulted in more than 7-fold increase of the thermostability compared to wild-type enzyme. AXE1dC-CBM22-2 also showed slightly higher thermostabilty than AXE1 (Fig. 3c). No significant differences were found in the pH stability of these enzymes. All of the enzymes had a broad range of pH tolerance (Fig. 3d).

Thermostability improvement by fusion with CBM4-2 and CBM22-2 might benefit from their thermophilic properties. CBM4-2 from the thermophilic bacterium *Rhodothermus marinus* Xyn10A had an estimated denaturation temperature of 87.4 °C, indicating it is a thermostable CBM [43]. CBM22-2 from *Clostridium thermocellum* Xyn10B had previously been described as a thermostabilizing domain on account of their apparent ability to confer thermostability to the catalytic module [44]. Thermostability improvement by fusion with thermostable CBM was previously reported for some cellulases and hemicellulases. For example, the chimeric enzyme comprising feruloyl esterase and CBM42 retained 40 % of its activity after incubation at 55 °C for 30 min, whereas, the native enzyme almost lost its activity [45]. Voutilainen et al. [46] reported that the adding of CBM1, CBM2 or CBM3 to the single-module cellobiohydrolase (Cel7A) from a thermophilic fungus *Talaromyces emersonii* increased the unfolding temperatures from 65 to 74 °C, 72 and 75 °C, respectively. Thermostable enzymes offer potential benefits in the hydrolysis of substrates, leading to improvement of the overall economy of the process [47].

Furthermore, fusion of CBM4-2 significantly enhanced the specific activity, the activity of AXE1dC-CBM4-2 towards 4-methylumbelliferyl acetate, was twice as high as AXE1. Kinetic parameters analysis also showed that the *V*_max_ and *K*_cat_/*K*_m_ value of AXE1dC-CBM4-2 was nearly two-fold higher compared to AXE1 (Table 1). But no significant difference of the specific activity was observed between AXE1 and other mutant enzymes (Table 1). It might be due to the intrinsic property of CBM4-2 or/and a favorable structural arrangement between the binding module and the catalytic domain [48].

**Table 1:** Specific activities and kinetic parameters of rAXE1s towards 4-methylumbelliferyl acetate

	Specific activity (IU/μmol)	*K*_m_ (μmol/L)	*V*_max_ (IU/μmol)	*K*_cat_ (s^−1^)	*K*_cat_/*K*_m_ (L · μmol^−1^ s^−1^)
AXE1dC	26,061 ± 469	86.79 ± 9.59	29,122 ± 676	485.4 ± 11.3	5.59 ± 1.17
AXE1	23,517 ± 177	86.76 ± 9.46	27,350 ± 626	455.8 ± 10.4	5.25 ± 1.09
AXE1dC-CBM4-2	45,708 ± 939	98.69 ± 14.15	53,842 ± 1718	897.4 ± 28.6	9.09 ± 2.02
AXE1dC-CBM6	22,652 ± 739	78.50 ± 9.85	29,080 ± 732	484.7 ± 12.2	6.17 ± 1.24
AXE1dC-CBM22-2	26,248 ± 550	94.04 ± 7.12	33,706 ± 504	561.8 ± 8.4	5.97 ± 1.17

### Binding specificity of rAXE1s

The ligand-binding properties of CBM1, 4-2, 6 and 22-2 had been characterized previously. CBM1 bound specifically to cellulose surfaces but not to mannan and xylan [49]. CBM4-2 showed high affinity for different soluble xylan, including birch xylan, wheat arabinoxylan, oat-spelt xylan and larchwood xylan, and phosphoric-acid swollen cellulose, but to Avicel very slightly [43, 50]. CBM6 had five sugar-binding sites and could accommodate highly decorated xylans but interacted weakly with soluble forms of cellulose [41, 51]. CBM22-2 had a cleft harboring a binding site for xylan. This protein could bind specifically to xylooligosaccharides and various substituted xylans, especially oat spelt xylan, wheat and rye arabinoxylan [42].

However, these substrates were restricted to purified polysaccharides that had an invariant chemical structure of β-1,4-linked xylose moieties. Here, three more complex substrates, including destarched wheat bran, NaClO_2_-treated wheat straw and water-insoluble wheat arabinoxylan (inAX), together with Avicel, were used in binding experiments to examine the specificity of CBMs. Table 2 gave the composition of three lignocellulose substrates.

**Table 2:** The moncomponent contents of different substrates

	Destarched wheat bran (%)	NaClO_2_-treated wheat straw (%)	InAX (%)
Acetyl groups	1.56 ± 0.14	3.70 ± 0.33	2.02 ± 0.08
Xylose	29.20 ± 1.64	27.02 ± 1.32	38.09 ± 2.37
Glucose	19.47 ± 1.79	43.15 ± 2.75	13.04 ± 1.22
Arabinose	19.00 ± 1.92	5.40 ± 0.34	25.56 ± 1.63
Lignin	14.65 ± 2.23	11.83 ± 1.52	7.89 ± 1.02

*InAX* water-insoluble wheat arabinoxylan

**Fig. 4:**
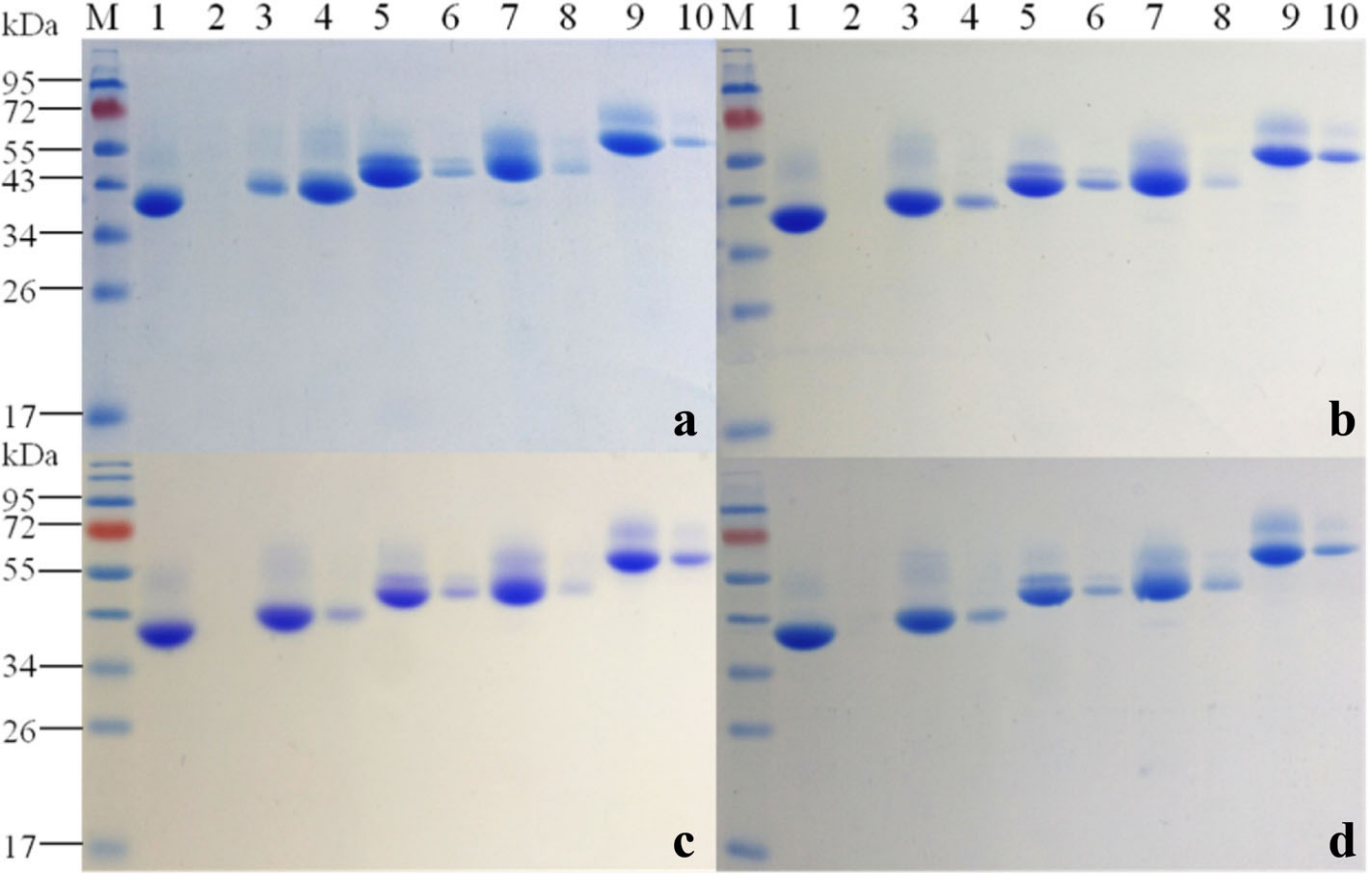
Binding specificities of rAXE1s to **a** Avicel, **b** destarched wheat bran, **c** NaClO_2_-treated wheat straw and **d** water-insoluble wheat arabinoxylan (inAX). Lanes: *M*, protein markers; *Lanes*: *1*, *3*, *5*, *7* and *9*, unbound fractions in supernatant, and *Lanes*: *2*, *4*, *6*, *8* and *10*, fractions bound to substrates for AXE1dC, AXE1, AXE1dC-CBM6, AXE1dC-CBM4-2, and AXE1dC-CBM22-2, respectively

Compared with earlier studies with pure xylans, AXE1dC-CBM4-2, AXE1dC-CBM6 and AXE1dC-CBM22-2 displayed a less capability to bind to substrates. As shown in Fig. 4, the proteins were mainly left in unbound fraction when rAXE1s were incubated with substrates at 0 °C for 1 h, except AXE1, which bound effectively to Avicel (Fig. 4a). AXE1dC was totally unable to bind to Avicel and other substrates. AXE1dC-CBM6 and AXE1dC-CBM22-2 bound slightly more to lignocellulose substrates than Avicel. Avicel is not pure cellulose but contains also xylan, which explained the binding of xylan-specific CBM variants with Avicel. However, AXE1dC-CBM4-2 could just bind to these four insoluble substrates very slightly. AXE1 also bound weakly to destarched wheat bran, NaClO_2_-treated wheat straw and inAX, probably due to simultaneous existence of cellulose in these substrates (Fig. 4b, c, d).

### Influence of different CBMs on the release of acetic acid from arabinoxylans

**Fig. 5:**
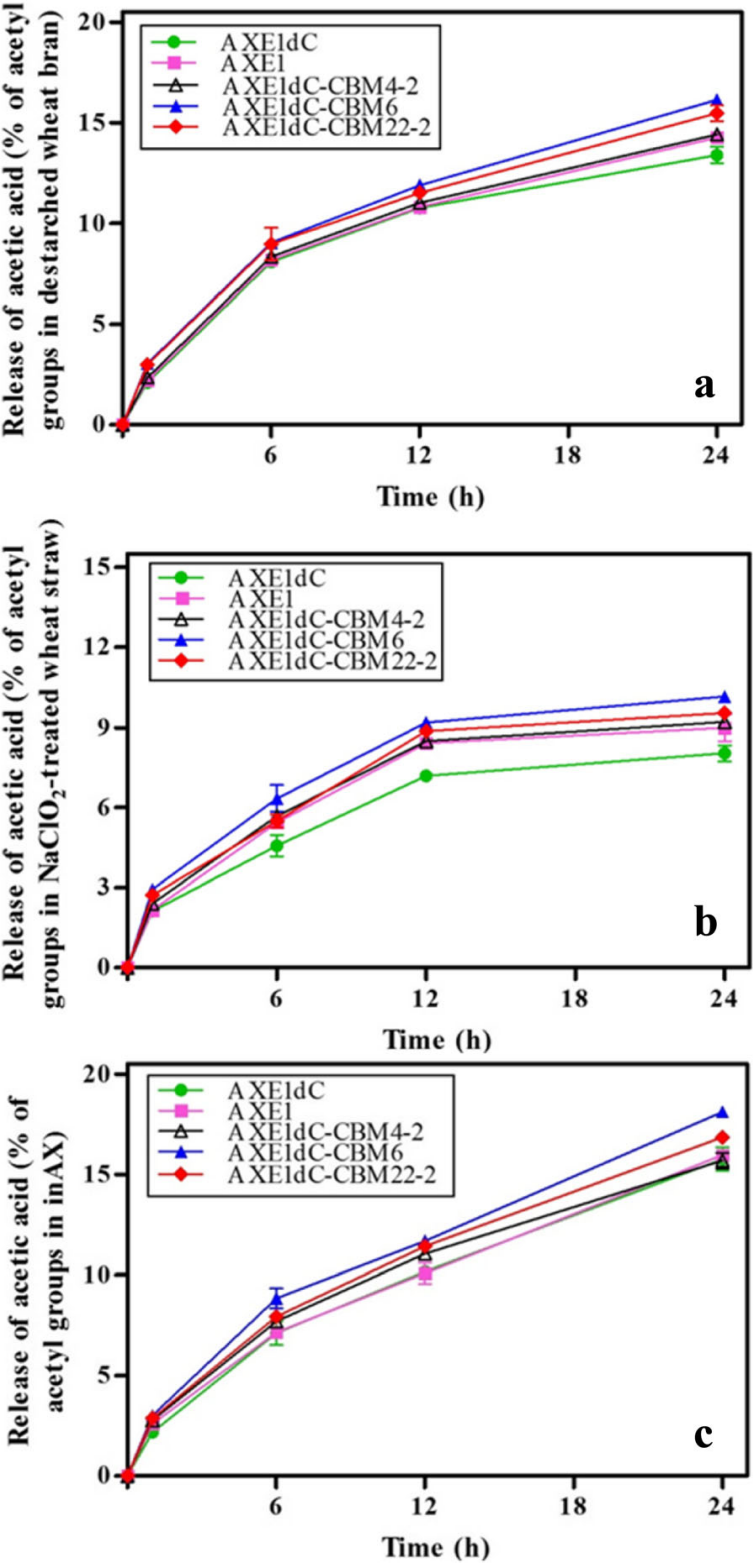
Time course for the enzymatic release of acetic acid from **a** destarched wheat bran, **b** NaClO_2_-treated wheat straw and **c** water-insoluble wheat arabinoxylan (inAX)

During the first hour, the release of acetic acid was very poor (below 3 %), but as time increased, it raised dramatically (Fig. 5). After a hydrolysis for 24 h, the cumulative release of acetic acid from inAX was the highest (over 15 %), followed by destarched wheat bran (about 14 %) and NaClO_2_-treated wheat straw (less than 10 %), although NaClO_2_-treated wheat straw was richest in acetyl groups (Table 3). Thus might be due to the low accessibility of acetyl groups in NaClO_2_-treated wheat straw. Overall, the release of acetic acid by rAXE1s in present study was much higher than that of AXE from *T. reesei* against hydrothermally pretreated wheat straw [10]. To some extent, the differences of pretreatment method of substrates and enzymes origins might cause the distinction of enzymatic hydrolysis yield.

**Table 3:** Percentage of released acetic acid from different substrates

Enzymes	Destarched wheat bran	NaClO_2_-treated wheat straw	InAX
	Released acetic acid (% total acetyl groups)	Released acetic acid (% total acetyl groups)	Release of acetic acid (% total acetyl groups)
AXE1dC	13.41 ± 0.56	8.04 ± 0.42	15.78 ± 0.85
AXE1	14.28 ± 0.14	9.00 ± 0.71	15.98 ± 0.42
AXE1dC-CBM4-2	14.38 ± 0.22	9.21 ± 0.31	15.80 ± 0.57
AXE1dC-CBM6	15.80 ± 0.17	10.17 ± 0.24	18.13 ± 1.01
AXE1dC-CBM22-2	15.49 ± 0.57	9.55 ± 0.51	16.87 ± 0.77

*InAX* water-insoluble wheat arabinoxylan

As expected, all rAXE1s with CBM displayed a higher capability in release of acetic acid from destarched wheat bran, NaClO_2_-treated wheat straw and inAX than AXE1dC, indicating the potentiation effect of CBM on rAXE1s activity. Furthermore, compared to AXE1, the replacement of CBM1 with CBM6 and CBM22-2, which bind to a broad range of xylans, resulted in 10.64, 13 and 13.45 %, and 8.47, 6.11 and 5.57 % more increases in release of acetyl groups from destarched wheat bran, NaClO_2_-treated wheat straw and inAX, respectively. AXE1dC-CBM4-2 displayed similar capacity in release of acetic acid as AXE1 (Table 3). CBMs can potentiate the appended carbohydrate-active enzyme action on lignocellulosic substrates via targeting and proximity effects depended on the sources of lignocellulosic substrates [13]. Mai-Gisondi et al. [52] investigated the ability of a family 3 cellulose-binding module (CBM3) fusion to enhance the activity of an acetylxylan esterase from *Aspergillus nidulans* (AnAXE, AN6093.2). It was found that the CBM3 fusion did not affect AnAXE catalytic efficiency on soluble substrates such as *p*-nitrophenyl acetate and 4-methylumbelliferyl acetate, or corncob xylan, but increased the activity by two to four times on cellulose acetate. The elevated deacetylating capacity of AXE1 was mainly attributed to the proximity effect of CBM1 because of its cellulose-specific binding property. Thus, CBM1 increased the concentration of AXE1 in the vicinity of the target substrate. On the other hand, the hydrolysis with AXE1dC-CBM4-2, AXE1dC-CBM6 and AXE1dC-CBM22-2 might be affected by targeting effect via assisting the enzyme attached directly to xylan. These results indicated the significance of targeting effect in AXE1 acetic acid release by xylan-specific CBMs, especially with CBM6 and CBM22-2.

### Influence of different CBMs on the synergistic actions between rAXE1s and XynII

**Fig. 6:**
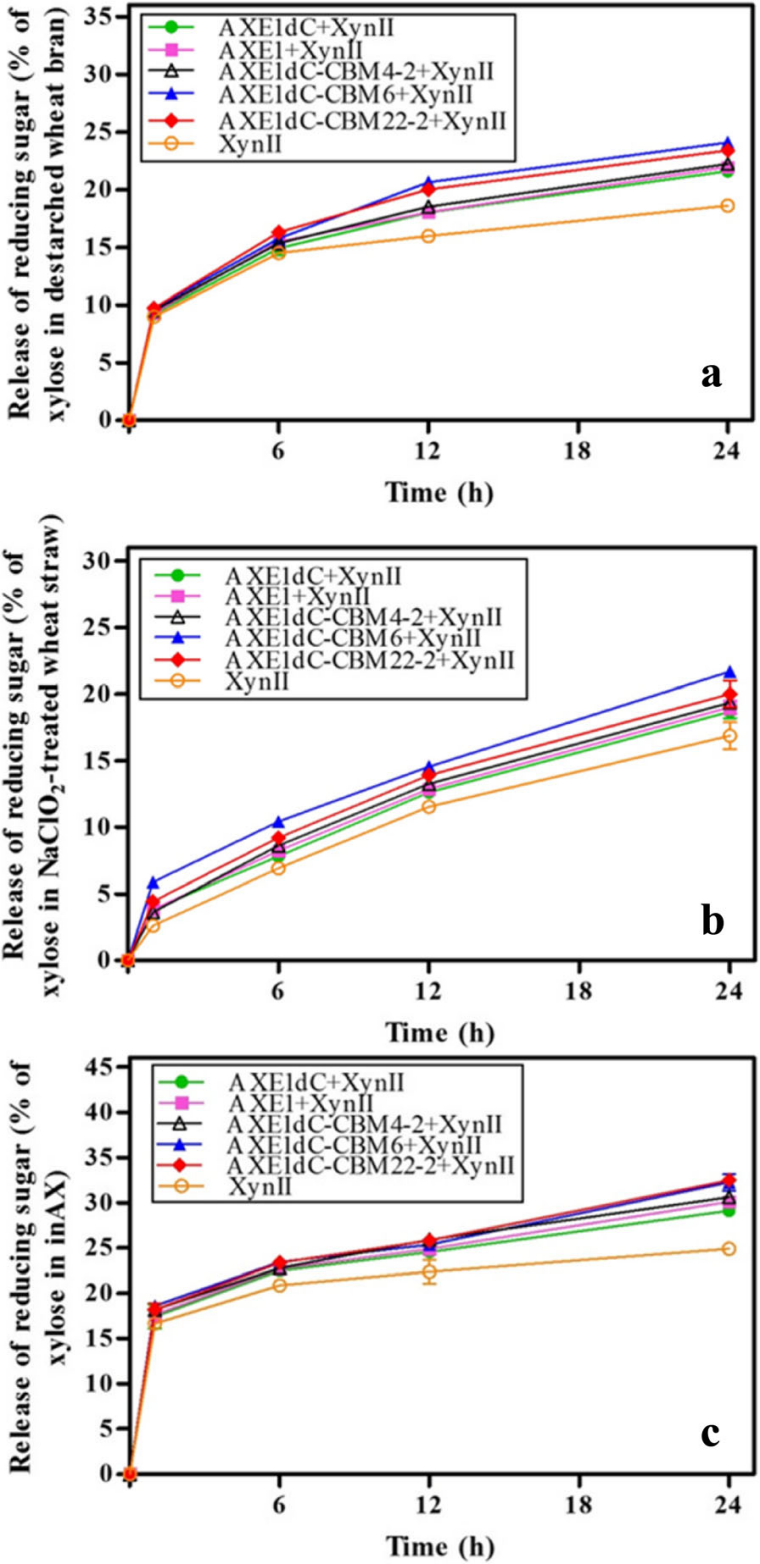
Time course for the release of reducing sugar from **a** destarched wheat bran, **b** NaClO_2_-treated wheat straw and **c** water-insoluble wheat arabinoxylan (inAX)

It could be seen that the combination of rAXE1s and XynII was more effective in each case than XynII alone (Fig. 6), implying the synergistic actions between rAXE1s and XynII against destarched wheat bran, NaClO2-treated wheat straw and water-insoluble wheat arabinoxylan (inAX) [53]. Overall, the release of total reducing sugar from inAX was the highest, followed by that of destarched wheat bran and NaClO_2_-treated wheat straw.

In general, the combination of rAXE1s with CBMs and XynII displayed a more obvious synergy in comparison to the combination of AXE1dC and XynII, implying the positive effect of CBMs on synergistic actions between rAXE1s and XynII against substrates. Moreover, AXE1dC-CBM6 and AXE1dC-CBM22-2 had a more positive impact on the synergistic actions than AXE1. The combination of AXE1dC-CBM6 and XynII was the highest, enhancing xylan hydrolysis by 29, 28, 34 % than XynII alone, which were 9.68, 9.73, 10.36 % more increases compared to the combination of AXE1 and XynII in the hydrolysis of destarched wheat bran, NaClO_2_-treated wheat straw and inAX for 24 h, respectively. The corresponding values for AXE1dC-CBM22-2 were 26, 24, 30 % more than XynII alone, and 6.61, 4.16, 7.9 % more increases compared to AXE1. However, there was no obvious distinction between AXE1dC-CBM4-2 and AXE1. The different binding specificities of CBMs belonging to different families might contribute to their difference in synergistic actions towards same substrates via targeting or proximity effects [51]. Since the xylan-specific CBMs, especially for CBM6 and CBM22-2, can target enzymes directly on the particular substrate xylan and potentiate the catalytic rates of AXE1dC-CBM6 and AXE1dC-CBM22-2, therefore increasing the efficiency of synergistic hydrolysis in comparison to AXE1 and AXE1dC.

**Fig. 7:**
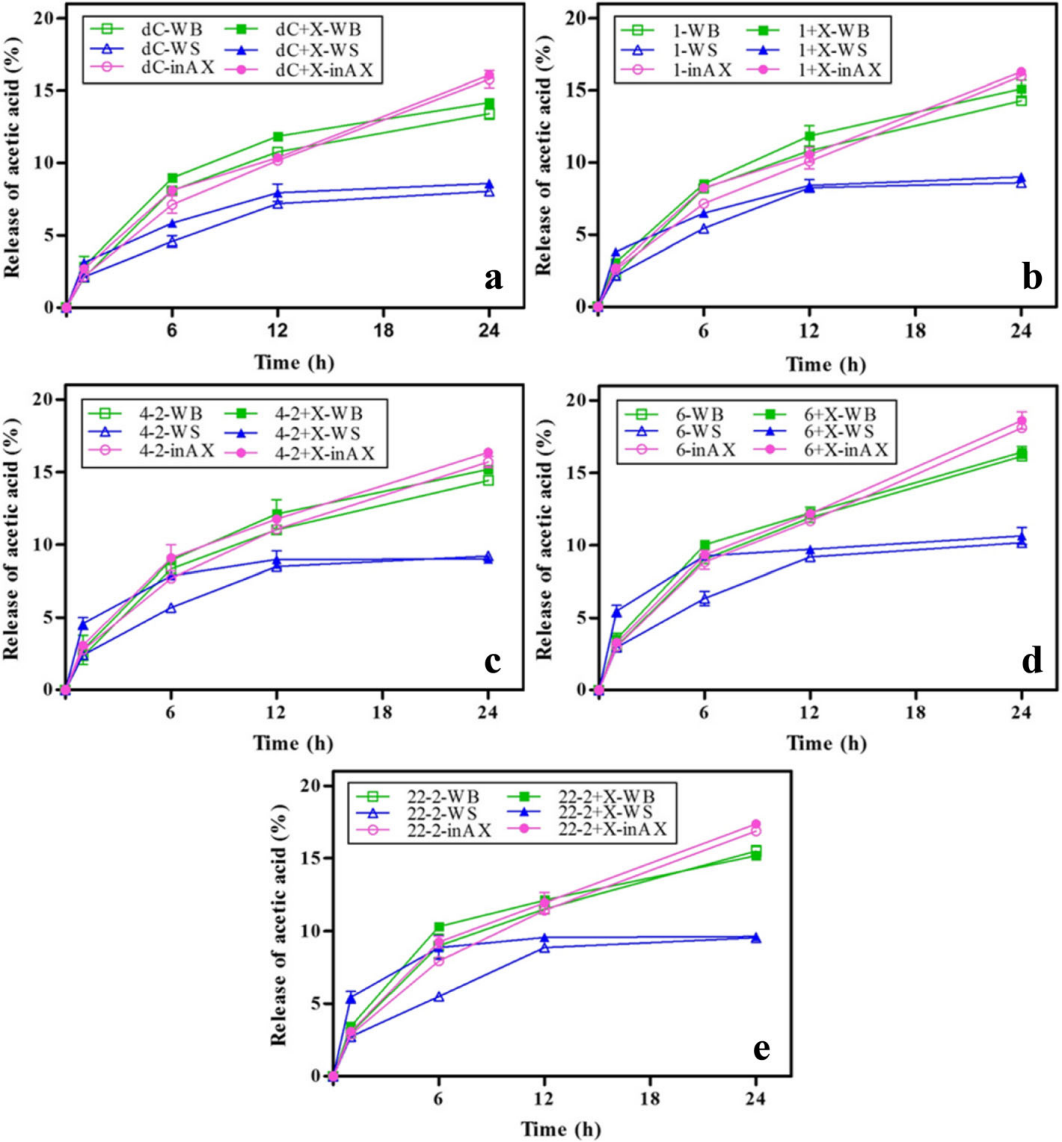
Release of acetic acid from destarched wheat bran (WB), NaClO_2_-treated wheat straw (WS) and water-insoluble wheat arabinoxylan (inAX) by simultaneous hydrolysis with XynII (X) and **a** AXE1dC (*dC*), **b** AXE1 (*1*), **c** AXE1dC-CBM4-2 (*4*-*2*), **d** AXE1dC-CBM6 (*6*) and **e** AXE1dC-CBM22-2 (*22*-*2*), respectively

The released acetic acid was also quantified in the synergism experiments. With the same rAXE1s enzyme loading, the adding of XynII improved the release of acetic acid as a whole (Fig. 7). As for destarched wheat bran and inAX, the releases of acetic acid were increased by about 15–17 % in each interval in synergism experiments than rAXE1 alone. However, a distinction of acetic acid release pattern existed between NaClO_2_-treated wheat straw with destarched wheat bran and inAX. The adding of XynII dramatically accelerated the release rate of acetic acid particularly in the first 12 h when NaClO_2_-treated wheat straw was used. Specially, the simultaneous action of XynII and AXE1dC-CBM4-2 increased the release of acetic acid by 90 and 38 % as compared to AXE1dC-CBM4-2 alone at the first and sixth hour. For synergism of XynII and AXE1dC-CBM6 or AXE1dC-CBM22-2, the corresponding values were 86, 46 and 100 %, 61 %, respectively. However, after 24 h, the improvement was not distinct between AXE1dC-CBM4-2 or AXE1dC-CBM22-2 alone with corresponding synergism with XynII. Perhaps, xylan-specific CBM4-2 and CBM22-2 targeted to the xylan in earlier stages when xylan in NaClO_2_-treated wheat straw was available, leading to accelerate the release of acetic acid during the first 12 h (Fig. 7c and e). But the targeting effect faded with the removal of xylan during the last 12 h [32].

The hydrolysis products of synergistic actions between rAXE1s and XynII were determined by HPAEC-PAD. The main products were X1 and X2 with tiny of X3. X2 was much higher than X1, whereas no X4 or above was detected. As shown in Table 4, rAXE1s with CBM6 and CBM22-2 caused the highest increases in the total amount of X1, X2 and X3 than other rAXE1s when simultaneous hydrolysis with XynII. This result was consistent well with the reducing sugar determined by Somogyi method (Fig. 6). It is interesting to note the ratios of X1 and X2 released from different substrates are different. Although the hemicellulose fraction of these three lignocellulose substrates is arabinoxylan, the structure and composition of arabinoxylan are still different, which may cause the different enzymatic hydrolysis efficiency and then the ratios of X1 and X2 from different substrates.

**Table 4:** The hydrolysis products of different substrates after 24 h hydrolysis by rAXE1s and XynII

		dC+ X	1+ X	4-2+ X	6+ X	22-2+ X	X
Destarched wheat bran	X1	11.3 ± 1.2	12.1 ± 1.9	12.8 ± 0.5	13.0 ± 1.2	12.9 ± 0.9	8.9 ± 0.7
	X2	18.5 ± 3.1	18.3 ± 2.0	17.8 ± 1.1	18.5 ± 1.1	18.4 ± 1.0	19.1 ± 0.9
	X3	0.2 ± 0.0	0.2 ± 0.0	0.2 ± 0.1	0.2 ± 0.0	0.2 ± 0.1	0.3 ± 0.0
	Total	30.0 ± 2.1	30.6 ± 1.9	30.8 ± 1.3	31.7 ± 1.1	31.5 ± 1.0	28.3 ± 0.9
NaClO_2_-treated wheat straw	X1	6.8 ± 1.3	7.4 ± 1.5	7.3 ± 0.9	7.9 ± 0.5	7.5 ± 1.8	6.0 ± 0.3
	X2	15.9 ± 2.4	15.5 ± 1.7	16.4 ± 2.1	16.8 ± 1.7	16.8 ± 1.7	14.6 ± 1.8
	X3	0.2 ± 0.0	0.2 ± 0.1	0.2 ± 0.1	0.2 ± 0.0	0.2 ± 0.0	0.6 ± 0.1
	Total	22.9 ± 1.6	23.1 ± 1.5	23.9 ± 1.6	24.9 ± 1.3	24.5 ± 1.7	21.2 ± 1.3
InAX	X1	8.2 ± 0.7	9.5 ± 0.9	10.1 ± 1.1	11.9 ± 1.2	10.0 ± 1.2	8.0 ± 1.1
	X2	25.5 ± 2.3	25.9 ± 1.8	25.9 ± 1.3	27.2 ± 1.5	29.9 ± 2.6	22.8 ± 2.2
	X3	0.2 ± 0.0	0.2 ± 0.0	0.2 ± 0.0	0.2 ± 0.0	0.2 ± 0.0	0.3 ± 0.0
	Total	33.9 ± 1.1	35.6 ± 1.4	36.2 ± 1.2	39.3 ± 1.4	40.1 ± 1.7	31.1 ± 1.4

The results are expressed as % of xylan (as xylose) in destarched wheat bran, NaClO_2_-treated wheat straw or water-insoluble wheat arabinoxylan (inAX). *dC* AXE1dC, *1* AXE1; *4*-*2* AXE1dC-CBM4-2, *6* AXE1dC-CBM6 *22*-*2* AXE1dC-CBM22-2; *X* XynII

**Fig. 8:**
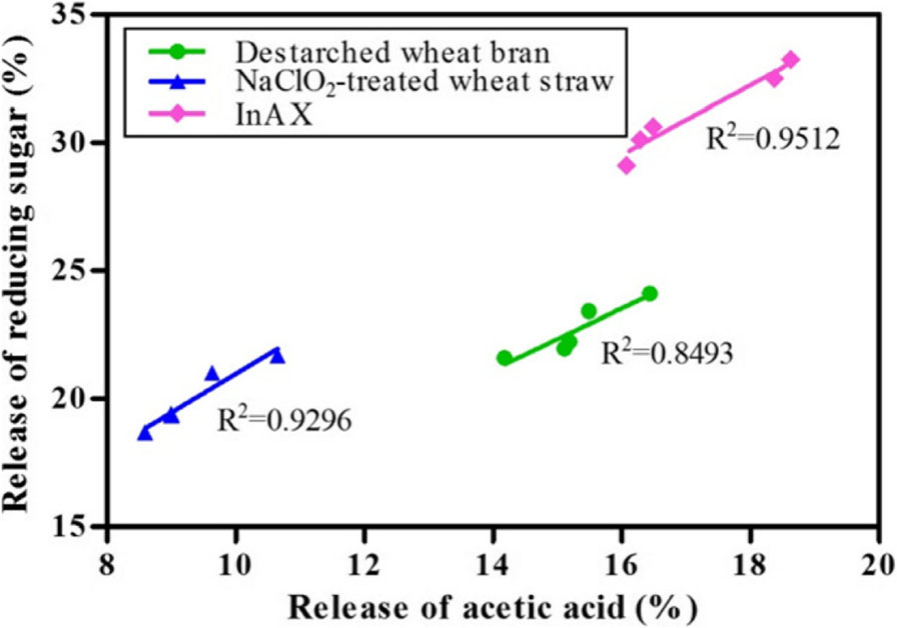
Linear relationships between release of acetic acid and reducing sugar from destarched wheat bran, NaClO_2_-treated wheat straw and water-insoluble wheat arabinoxylan (inAX) after 24 h hydrolysis by combined rAXE1s and XynII

A high linear correlation exists between the release of acetic acid and reducing sugar (Fig. 8). The *R* ^2^ of destarched wheat bran, NaClO_2_-treated wheat straw and inAX was 0.8493, 0.9296 and 0.9512, respectively. This suggested that the removal of acetyl groups contributed to the release of xylose from xylans. For the synergistic actions between rAXE1s and XynII, the highest enhancement was obtained in inAX due to the highest release of acetic acid as compared with destarched wheat bran and NaClO_2_-treated wheat straw. This suggested that the synergy was more pronounced in the materials containing a high accessibility of acetyl groups. These results were in good agreement with reports on the combination of endoxylanase and AXE in increasing the xylan conversion, and consequently the cellulose conversion to glucose by cellulolytic enzymes [3, 54].

## Conclusion

Cellulose-specific CBM1 in AXE1 obviously contributes to its catalytic action against lignocellulosic substrates compared with AXE1dC. However, the replacement of CBM1 with xylan-specific CBM4-2 significantly enhanced AXE1 thermostability and catalytic activity against soluble substrate 4-methylumbelliferyl acetate. Whereas replacement with xylan-specific CBM6 and CBM22-2 were more effective in enzymatic release of acetic acid from destarched wheat bran, NaClO_2_-treated wheat straw, and water-insoluble wheat arabinoxylan compared to AXE1. Moreover, replacement with CBM6 and CBM22-2 also resulted in higher degree releases of xylose and acetic acid from different lignocellulosic substrates when simultaneous hydrolysis with xylanase. A good linear relationship exists between acetic acid and the reducing sugar release. Our findings suggested the significance of targeting effect in AXE1 catalysis against lignocellulosic substrates mediated by xylan-specific CBMs.

## Methods

### Gene, strains and chemicals

The plasmid pPICZαA-*axe1*, which has been previously constructed to express mature AXE1 [39], was used as a template for recombinant acetyl xylan esterases construction. The gene sequences encoding xylan-specific CBM4-2, CBM6, and CBM22-2 modules were originally from *Rhodothermus marinus* Xyn10A, *Clostridium thermocellum* Xyn11A, and *Clostridium thermocellum* Xyn10B, respectively (Additional file 1) [40–42]. The corresponding gene fragments were synthesized by Springen Biotech Co. (Jiangsu, China) and carried into plasmids pUC57 with modified codon according to the codon preference of *Pichia pastoris. Escherichia coli* DH5α was used for plasmid construction and propagation. *P. pastoris* KM71H and the plasmid pPICZαA from Invitrogen (Carlsbad, CA, USA) were used for heterologous expression.

The substrates 4-methylumbelliferyl acetate and Avicel were purchased from Sigma-Aldrich (St. Louis, MO). Wheat bran was obtained from Yongfang (Shandong, China) and wheat straw was obtained from Lian Yungang (Jiangsu, China). Water-insoluble wheat arabinoxylan (inAX) was purchased from Megazyme (P-WAXYI, Wicklow Ireland). TransStart FastPfu DNA polymerase and T4 DNA ligase were purchased from TransGen Biotech (Beijing, China). The restriction enzymes and endoglycosidase H were purchased from New England Biolabs (Beverly, MA, USA). All other chemicals used were analytical grade.

### Construction of recombinant plasmids

The *cbm1*-deleted derivative, *axe1dC*, was amplified by the polymerase chain reaction (PCR) using pPICZαA-*axe1* as template and the *axe1dC* F1 forward primer and *axe1dC* R1 reverse primer (Additional file 2: Table S1). *Eco*RI and *Not*I restriction enzyme sites were introduced to the 5′ and 3′ of *axe1dC* DNA fragment. After digestion, the fragment was ligated at the *Eco*RI/*Not*I sites of pPICZαA Pichia expression vector to yield the expression plasmid pPICZαA-*axe1dC*. Three CBM-fused derivatives, *axe1dC*-*cbm4-2*, *axe1dC*-*cbm6*, *and axe1dC*-*cbm22-2* were created by combining *axe1dC* with the fragment encoding CBM4-2, CBM6 and CBM22-2, respectively. The fragment encoding catalytic domain and linker, *axe1dC*, was amplified by PCR using pPICZαA-*axe1* as template and the *axe1dC* F2 forward primer and *axe1dC* R2 reverse primer. *Eco*RI and *Sac*II restriction enzyme sites were introduced to the 5′ and 3′ of *axe1dC* DNA fragment. The *cbm4-2* DNA fragment was amplified from pUC57-*cbm4-2* using the *cbm4-2* F3 forward primer and *cbm4-2* R3 reverse primer. The *cbm6* DNA fragment was amplified from pUC57-*cbm6* using the *cbm6* F4 forward primer and *cbm6* R4 reverse primer. And the *cbm22-2* DNA fragment was amplified from pUC57-*cbm22-2* using the *cbm22-2* F5 forward primer and *cbm22-2* R5 reverse primer. *Sac*II and *Not*I restriction enzyme sites were introduced to the 5′ and 3′ of *cbm4-2*, *cbm6* and *cbm22-2* DNA fragment, respectively. After digestion, the fragments *axe1dC* and *cbm4-2*, *cbm6*, *cbm22-2* were ligated at the *Eco*RI/*Not*I sites of pPICZαA to yield the plasmids pPICZαA-*axe1dC*-*cbm4-2*, pPICZαA-*axe1dC*-*cbm6*, pPICZαA-*axe1dC*-*cbm22-2*, respectively (Additional file 3: Figure S1). All rAXE1s, including wild-type enzyme AXE1, were fused with a 6-histidine tag at the C-terminus to facilitate purification using affinity chromatography.

### Expression, purification and molecular weight analysis

The plasmids were linearized using *Sac*I and transformed into *Pichia pastoris* KM71H competent cells by electroporation according to the *Pichia* expression system manual from Invitrogen. All rAXE1s, including AXE1, were produced by methanol induction at a final concentration of 2.5 % according to the method described previously [55]. Supernatants from 25 mL cultures were collected by centrifugation (5000 g for 15 min) and then the crude enzymes were purified by affinity chromatography using Ni-NTA Agarose gel (Qiagen, Valencia, CA, USA) according to the manufacturer’s manual. The enzyme homogeneity and molecular weights of purified enzymes were estimated using sodium dodecyl sulfate-polyacrylamide gel electrophoresis (SDS-PAGE) [12 % (*w/v*)]. Purified rAXE1s were deglycosylated with endoglycosidase H and analyzed by SDS-PAGE. XynII was produced and purified as described previously [53].

### Specific activity and biochemical properties of rAXE1s towards soluble substrate

The rAXE1s activity were determined spectrophotometrically at 50 °C by measuring the increasing in A_354_ nm during the initial 1 min of the assay resulting from the release of 4-methylumbelliferone from 4-methylumbelliferyl acetate [56]. Reaction mixtures consisted of: 1398 μL 1 M potassium phosphate buffer (pH 8.0), 100 μL 10 mM 4-methylumbelliferyl acetate and 2 μL purified enzyme. One unit of enzyme activity (IU) was defined as the quantity of enzyme required to release 1 μmol of 4-methylumbelliferone per minute.

Optimal pH and temperature values were determined over the ranges pH 6.0–9.0 (universal buffer: 50 mM H_3_PO_4_, 50 mM CH_3_COOH, 50 mM H_3_BO_3_, pH adjusted by 0.2 M NaOH at 25 °C) and 40–60 °C, respectively. To determine pH stability, rAXE1s were incubated at different buffer (pH 3.0–11.0) at 4 °C for 12 h. Thermostability of rAXE1s were determined by being incubated at 55 °C for 0–90 min. Residual activities towards 4-methylumbelliferyl acetate were compared with control samples. The kinetic constants for rAXE1s were assayed by measuring the rate of 4-methylumbelliferyl acetate hydrolysis under the standard assay conditions using a substrate concentration range of 0.33–1.33 mM.

### Substrate composition analysis

The preparation of destarched wheat bran and NaClO_2_-treated wheat straw was described by Wang et al. [57] and Saarnio et al. [58, 59], respectively. The carbohydrate and lignin content of destarched wheat bran, NaClO_2_-treated wheat straw and inAX was determined by the standard procedure of NREL [60]. The acetic acid content was determined after alkaline hydrolysis (2 M NaOH, 4 h, 70 °C) [61] and quantified by the Acetic Acid Assay Kit (Megazyme) according to the manufacturer’s instructions.

### Binding specificity of rAXE1s

Since the optimal pH of xylanase used in synergistic hydrolysis was 7.0, the substrate binding and hydrolysis experiment on insoluble substrates were both carried out at pH 7.0. Reaction mixtures (2 mL) containing 50 mg substrate and 250 μg purified rAXE1s in pre-cooled 100 mM potassium phosphate buffer (pH 7.0) were incubated on a rotating shaker (200 r.p.m.) at 0 °C for 60 min. After incubation, samples were centrifuged (10,000 g, 4 °C) for 3 min. The unbound proteins in the supernatant were concentrated by PEG6000. The precipitations were washed three times with pre-cooled 100 mM potassium phosphate buffer (pH 7.0). The unbound and bound proteins in the supernatants and precipitates, respectively, were boiled with SDS-sample buffer for 5 min and analyzed by SDS-PAGE analysis.

### Influence of different CBMs on the enzymatic release of acetic acid from arabinoxylans

Reaction mixtures consisted of: 0.1 g destarched wheat bran, NaClO_2_-treated wheat straw or inAX [2 % (*w/v*) suspension in 100 mM sodium citrate buffer, pH 7.0] with 0.005 μmol of purified enzyme in a total volume of 5 mL. Mixtures were incubated at 50 °C for 1, 6, 12, 24 h with orbital shaking (150 r.p.m.) and then boiled at 99 °C for 10 min. All hydrolysis experiments were carried out in duplicates. After centrifugation, the acetic acid in the supernatant was quantified using the Acetic Acid Assay Kit (Megazyme) according to the manufacturer’s instructions.

### Influence of different CBMs on the synergistic actions between rAXE1s and XynII

The synergy between recombinant acetyl xylan esterases and xylanase in the hydrolysis of the destarched wheat bran, NaClO_2_-treated wheat straw and inAX, was carried out at pH 7.0 and 50 °C. rAXE1s (100 IU) and XynII (25 IU) were added simultaneously to the reaction mixtures containing 0.1 g substrate and a moderate amount of 100 mM sodium citrate buffer (pH 7.0) in a total volume of 5 mL. A final concentration of 25 mg/L Ampicillin and Zeocin were added to prevent the reducing sugar consumption of microbes. Mixtures were incubated at 50 °C for 1, 6, 12, 24 h with orbital shaking (150 r.p.m.) and then boiled at 99 °C for 10 min. The acetic acid and reducing sugar released were quantified using the Acetic Assay Kit and Somogyi-Nelson method with xylose as standard, respectively. Xylose and xylooligosaccharides in the hydrolysates were analyzed at 25 °C using a Carbo-Pac PA200 column (3 × 250 mm) fitted to an ICS-3000 high-performance anion exchange chromatography system (Dionex, Sunnyvale, CA, USA) with pulsed amperometric detection (HPAEC-PAD). A dual mobile-phase system (A, 100 mM NaOH; B, 500 mM sodium acetate) was applied, and saccharides were eluted using a linear sodium acetate gradient (B:0–24 % in 40 min; 0.3 ml/min), followed by elution with 100 mM NaOH (15 min; 0.3 ml/min) as previously described [53].

## Additional files

Additional file 1: Sequences of CBMs and rAXE1s. (DOC 44 kb)
Additional file 2: Table S1. Nucleotide sequence of the primers used in amplification reactions. (DOC 35 kb)
Additional file 3: Figure S1. Construction of recombinant plasmids. (DOC 57 kb)

## Abbreviations

AXE1: Acetyl xylan esterase 1 from *Volvariella volvacea*
AXE1dC: CBM1-deleted acetyl xylan esterase 1
AXE1dC-CBM22-2: Fusion of CBM1-deleted acetyl xylan esterase 1 and the second family 22 carbohydrate binding module from *Clostridium thermocellum* xylanase 10B
AXE1dC-CBM4-2: Fusion of CBM1-deleted acetyl xylan esterase 1 and the second family 4 carbohydrate binding module from *Rhodothermus marinus* xylanase 10A
AXE1dC-CBM6: Fusion of CBM1-deleted acetyl xylan esterase 1 and family 6 carbohydrate binding module from *Clostridium thermocellum* xylanase 11A
CBM: Carbohydrate binding module
HPAEC-PAD: High-performance anion exchange chromatography coupled with pulsed amperometric detection
inAX: Water-insoluble wheat arabinoxylan
PEG6000: Polyethylene Glycol 6000
rAXE1s: Recombinant AXE1s including AXE1dC, AXE1, AXE1dC-CBM4-2, AXE1dC-CBM6, AXE1dC-CBM22-2
XynII: Xylanase II from *Volvariella volvacea*
