# Supplementary material for: Replacement of carbohydrate binding modules improves acetyl xylan esterase activity and its synergistic hydrolysis of different substrates with xylanase
Source: BMC Biotechnol. 2016 Oct 22;16:73. doi: 10.1186/s12896-016-0305-6 (PMC5075172; doi:10.1186/s12896-016-0305-6)
Supplement: Additional file 1: — Sequences of CBMs and rAXE1s. (DOC 44 kb) [file 12896_2016_305_MOESM1_ESM.doc]

**Sequences of CBMs and** **rAXE1s**

1.The original sequences of CBM4-2, CBM6, CBM22-2

Table S2 The origin of CBMs

| CBM | Origin | Organism | GenBank ID | Binding site |
| --- | --- | --- | --- | --- |
| CBM4-2 | Xyn10A | *Rhodothermus marinus* | CAA72323.2 | N-terminal |
| CBM6 | Xyn11A | *Clostridium thermocellum* | AAC04579.1 | C-terminal |
| CBM22-2 | Xyn10B | *Clostridium thermocellum* | AAR39814.1 | C-terminal |

**CBM4-2**

gagcttgtcgccaacatcaacggtggattcgaatcgacgccggccggggtggtgacggatctggccgaaggtgtggagggctgggatctgaacgtgggctcctcggtgacgaatccgccggtttttgaagtgctggagacgtccgatgcccctgaagggaataaggtgctggcggtgacggtcaacggggtgggcaacaacccctgggacatcgaggcgacggccttcccggtgaacgtacgtccgggcgtgacctacacctacacgatctgggcgcgggccgagcaggacggggcggtggtcagcttcacggtggggaaccagtcgttccaggagtacgggcgactgcatgagcagcagatcacgaccgagtggcagccgttcacgttcgagtttacggtcagtgatcaggagacggtcattcgggcgccgatccattttggctatgcggccaacgtcggcaataccatctacattgatggcctggccattgtggac

166 aa

ELVANINGGFESTPAGVVTDLAEGVEGWDLNVGSSVTNPPVFEVLETSDAPEGNKVLAVTVNGVGNNPWDIEATAFPVNVRPGVTYTYTIWARAEQDGAVVSFTVGNQSFQEYGRLHEQQITTEWQPFTFEFTVSDQETVIRAPIHFGYAANVGNTIYIDGLAIVD

**CBM6**

agaagtgctttttcaaaaatcgaatctgaggagtacaactccctcaagtcatcaaccattcagaccataggcacttccgacggaggaagcggtataggttatattgaaagcggtgactatctggtatttaacaaaataaactttggaaacggtgcaaactctttcaaggcaagggttgcatccggtgcggacacacccaccaatatccagttaagactcggaagcccgaccggtactcttataggaactcttacggtggcttccacaggtggttggaacaattacgaggaaaaatcctgcagcataaccaacactacaggacagcacgacttatatctggtattctcaggtcctgttaacattgactacttcatattcgactcgaatggcgtaaat

132 aa

RSAFSKIESEEYNSLKSSTIQTIGTSDGGSGIGYIESGDYLVFNKINFGNGANSFKARVASGADTPTNIQLRLGSPTGTLIGTLTVASTGGWNNYEEKSCSITNTTGQHDLYLVFSGPVNIDYFIFDSNGVN

**CBM22-2**

aaaccggaagagccggatgcaaacggatattattatcatgacacttttgaaggaagcgtaggacagtggacagccagaggacctgcggaagttctgcttagcggaagaacggcttacaaaggttcagaatcactcttggtaaggaaccgtacggcagcatggaacggagcacaacgggcgctgaatcccagaacgtttgttcccggaaacacatattgtttcagcgtagtggcatcgtttattgaaggtgcgtcttccacaacattctgcatgaagctgcaatacgtagacggaagcggcactcaacggtatgataccatagatatgaaaactgtgggtccaaatcagtgggttcacctgtacaatccgcaatacagaattccttccgatgcaacagatatgtatgtttatgtggaaacagcggatgacaccattaacttctacatagatgaggcaatcggagcggttgccggaactgtaatcgaagga

163 aa

KPEEPDANGYYYHDTFEGSVGQWTARGPAEVLLSGRTAYKGSESLLVRNRTAAWNGAQRALNPRTFVPGNTYCFSVVASFIEGASSTTFCMKLQYVDGSGTQRYDTIDMKTVGPNQWVHLYNPQYRIPSDATDMYVYVETADDTINFYIDEAIGAVAGTVIEG

2. The optimized (*Pichia pastoris*) sequences of of rAXE1s

AXE1dC

gaattccaattgcaacaggttactaactttggacctaacccaactaacgtcggtatctacgcctacagaccatccggtttgccagccaatcctgctttgatcgttgccatgcattactgcaccggtaccgctcaagcttactactctggtaccagatacgctactcttgctaacacttacaagttcattgttatttaccctaacgctccagactctggtggatgctgggatgtccacaccaacgctactttgactcacgacgccggtggagactccttgggtatcgcttctgctatcagatacgccattaacacttggggtgtcgacagaaatcgtgttttcgctactggtacctcttccggagccatgatgactaacgtcttggctggtgcttaccctgacttgattaaggctggtgctgcctttgctggtgttccttacggttgtttcgccggtccaggtatgtggaactctcagtgtgctcagggtcagcttactaagactgcccagcaatggggtgaccaagtccgttctggttaccctggatacaccggatccagacctaagatgcagatctggcacggtaccagagatgaaactttgaattacaataacttcgacgaagctattaaacagtggaccaatatcttcggatactccactaccgctacccaagtccaacagaactccccactttctggttggactcgttccacttacggaccaaacttccaagctatttctgctgctaacgttccacacaatattccagttcaagagaacgacgtccttaactggttcggaattactggtggaggtccaactaccactaccactaccactggaccaacccaacctccaaccactaccactcctggtggacctcatcatcatcatcatcattgagcggccgc

*Note*: Restriction enzyme sites are underlined. Catalytic domain is colored black. Linker is colored green. 6×His tag and terminator codon are colored blue.

AXE1

gaattccaattgcaacaggttactaactttggacctaacccaactaacgtcggtatctacgcctacagaccatccggtttgccagccaatcctgctttgatcgttgccatgcattactgcaccggtaccgctcaagcttactactctggtaccagatacgctactcttgctaacacttacaagttcattgttatttaccctaacgctccagactctggtggatgctgggatgtccacaccaacgctactttgactcacgacgccggtggagactccttgggtatcgcttctgctatcagatacgccattaacacttggggtgtcgacagaaatcgtgttttcgctactggtacctcttccggagccatgatgactaacgtcttggctggtgcttaccctgacttgattaaggctggtgctgcctttgctggtgttccttacggttgtttcgccggtccaggtatgtggaactctcagtgtgctcagggtcagcttactaagactgcccagcaatggggtgaccaagtccgttctggttaccctggatacaccggatccagacctaagatgcagatctggcacggtaccagagatgaaactttgaattacaataacttcgacgaagctattaaacagtggaccaatatcttcggatactccactaccgctacccaagtccaacagaactccccactttctggttggactcgttccacttacggaccaaacttccaagctatttctgctgctaacgttccacacaatattccagttcaagagaacgacgtccttaactggttcggaattactggtggaggtccaactaccactaccactaccactggaccaacccaacctccaaccactaccactcctggtggacctttgcaaactcaatggggtcaatgtggtggtatcggttggaccggtccaactgcttgccaaccaccattcacttgtcaatacaccaatgactggtactctcagtgtagacatcatcatcatcatcattgagcggccgc

Note: Restriction enzyme sites are underlined. Catalytic domain is colored black. Linker is colored green. CBM1 is colored red. 6×His tag and terminator codon are colored blue.

AXE1dC-CBM4-2

gaattccaattgcaacaggttactaactttggacctaacccaactaacgtcggtatctacgcctacagaccatccggtttgccagccaatcctgctttgatcgttgccatgcattactgcaccggtaccgctcaagcttactactctggtaccagatacgctactcttgctaacacttacaagttcattgttatttaccctaacgctccagactctggtggatgctgggatgtccacaccaacgctactttgactcacgacgccggtggagactccttgggtatcgcttctgctatcagatacgccattaacacttggggtgtcgacagaaatcgtgttttcgctactggtacctcttccggagccatgatgactaacgtcttggctggtgcttaccctgacttgattaaggctggtgctgcctttgctggtgttccttacggttgtttcgccggtccaggtatgtggaactctcagtgtgctcagggtcagcttactaagactgcccagcaatggggtgaccaagtccgttctggttaccctggatacaccggatccagacctaagatgcagatctggcacggtaccagagatgaaactttgaattacaataacttcgacgaagctattaaacagtggaccaatatcttcggatactccactaccgctacccaagtccaacagaactccccactttctggttggactcgttccacttacggaccaaacttccaagctatttctgctgctaacgttccacacaatattccagttcaagagaacgacgtccttaactggttcggaattactggtggaggtccaactaccactaccactaccactggaccaacccaacctccaaccactaccactcctggtggacctccgcgggagttggtcgccaacatcaacggtggtttcgagtccaccccagccggtgtcgtcactgacttggccgagggtgtcgagggttgggacttgaacgtcggttcctccgtcaccaacccacctgtcttcgaggtcttggagacctccgacgctccagagggtaacaaggtcttggccgtcaccgtcaacggtgtcggaaacaacccatgggacatcgaggccaccgcctttccagttaacgtccgtcctggagtcacctacacctacaccatctgggccagagccgagcaggacggagctgttgtctccttcaccgtcggtaaccagtccttccaggagtacggacgtttgcacgagcagcagatcaccaccgagtggcaaccattcaccttcgagttcaccgtctccgaccaggaaaccgtcatccgtgccccaatccacttcggttatgccgccaacgtcggaaacaccatctacatcgacggattggctatcgttgaccatcatcatcatcatcattgagcggccgc

Note: Restriction enzyme sites are underlined. Catalytic domain is colored black. Linker is colored green. CBM4-2 is colored red. 6×His tag and terminator codon are colored blue.

AXE1dC-CBM6

gaattccaattgcaacaggttactaactttggacctaacccaactaacgtcggtatctacgcctacagaccatccggtttgccagccaatcctgctttgatcgttgccatgcattactgcaccggtaccgctcaagcttactactctggtaccagatacgctactcttgctaacacttacaagttcattgttatttaccctaacgctccagactctggtggatgctgggatgtccacaccaacgctactttgactcacgacgccggtggagactccttgggtatcgcttctgctatcagatacgccattaacacttggggtgtcgacagaaatcgtgttttcgctactggtacctcttccggagccatgatgactaacgtcttggctggtgcttaccctgacttgattaaggctggtgctgcctttgctggtgttccttacggttgtttcgccggtccaggtatgtggaactctcagtgtgctcagggtcagcttactaagactgcccagcaatggggtgaccaagtccgttctggttaccctggatacaccggatccagacctaagatgcagatctggcacggtaccagagatgaaactttgaattacaataacttcgacgaagctattaaacagtggaccaatatcttcggatactccactaccgctacccaagtccaacagaactccccactttctggttggactcgttccacttacggaccaaacttccaagctatttctgctgctaacgttccacacaatattccagttcaagagaacgacgtccttaactggttcggaattactggtggaggtccaactaccactaccactaccactggaccaacccaacctccaaccactaccactcctggtggacctccgcggagatccgccttctccaagattgaatctgaagagtacaactctcttaaatcttccactattcaaactattggaacctccgacggtggatccggtattggttacattgaatccggagactacttggttttcaataagattaactttggtaacggtgctaattctttcaaggctagagtcgcttctggagccgacaccccaactaacattcagttgagattgggttctccaaccggtactttgatcggtaccttgactgttgcttctactggtggatggaacaattacgaggaaaagtcctgttctatcaccaacactactggtcaacacgacttgtaccttgttttctctggtccagtcaacatcgactacttcatctttgattctaacggagtcaatcatcatcatcatcatcattgagcggccgc

Note: Restriction enzyme sites are underlined. Catalytic domain is colored black. Linker is colored green. CBM6 is colored red. 6×His tag and terminator codon are colored blue.

AXE1dC-CBM22-2

gaattccaattgcaacaggttactaactttggacctaacccaactaacgtcggtatctacgcctacagaccatccggtttgccagccaatcctgctttgatcgttgccatgcattactgcaccggtaccgctcaagcttactactctggtaccagatacgctactcttgctaacacttacaagttcattgttatttaccctaacgctccagactctggtggatgctgggatgtccacaccaacgctactttgactcacgacgccggtggagactccttgggtatcgcttctgctatcagatacgccattaacacttggggtgtcgacagaaatcgtgttttcgctactggtacctcttccggagccatgatgactaacgtcttggctggtgcttaccctgacttgattaaggctggtgctgcctttgctggtgttccttacggttgtttcgccggtccaggtatgtggaactctcagtgtgctcagggtcagcttactaagactgcccagcaatggggtgaccaagtccgttctggttaccctggatacaccggatccagacctaagatgcagatctggcacggtaccagagatgaaactttgaattacaataacttcgacgaagctattaaacagtggaccaatatcttcggatactccactaccgctacccaagtccaacagaactccccactttctggttggactcgttccacttacggaccaaacttccaagctatttctgctgctaacgttccacacaatattccagttcaagagaacgacgtccttaactggttcggaattactggtggaggtccaactaccactaccactaccactggaccaacccaacctccaaccactaccactcctggtggacctccgcggaagcctgaagagccagacgccaacggatactactaccatgacaccttcgagggttctgtcggtcagtggaccgccagaggtccagccgaggtcttgttgtccggacgtaccgcctacaagggatccgagtccttgttggtccgtaaccgtactgccgcctggaacggtgctcagagagcccttaacccacgtaccttcgtcccaggtaacacctactgcttctccgtcgttgcctccttcatcgagggagcctcctccactaccttctgcatgaagcttcagtacgtcgacggttccggaacccaacgttacgacaccatcgacatgaagaccgtcggtccaaaccagtgggtccacttgtacaacccacagtaccgtatcccatccgatgccaccgacatgtacgtctacgttgagaccgccgacgacaccatcaacttctacatcgacgaggccatcggtgccgttgccggtaccgttatcgagggtcatcatcatcatcatcattgagcggccgc

Note: Restriction enzyme sites are underlined. Catalytic domain is colored black. Linker is colored green. CBM22-2 is colored red. 6×His tag and terminator codon are colored blue.
