# Supplementary material for: Replacement of carbohydrate binding modules improves acetyl xylan esterase activity and its synergistic hydrolysis of different substrates with xylanase
Source: BMC Biotechnol. 2016 Oct 22;16:73. doi: 10.1186/s12896-016-0305-6 (PMC5075172; doi:10.1186/s12896-016-0305-6)
Supplement: Additional file 2: Table S1. — Nucleotide sequence of the primers used in amplification reactions. (DOC 35 kb) [file 12896_2016_305_MOESM2_ESM.doc]

Table S1 Nucleotide sequence of the primers used in amplification reactions

| Primer | Sequence(5’-3’) | Restriction site^a^ |
| --- | --- | --- |
| *axe1dC* F1 (*axe1dC* F2) | GGAATTCCAATTGCAACAGGTTACTAA | *Eco*RI |
| *cbm4-2* F3 | TCCCCGCGGGAGTTGGTCGCCAACATCAACG | *Sac*II |
| *cbm* *6* F4 | TCCCCGCGGAGATCCGCCTTCTCCAAGATTG | *Sac*II |
| *cbm* *22-2* F5 | TCCCCGCGGAAGCCTGAAGAGCCAGACGC | *Sac*II |
| *axe1dC* R1 | ATAAGAATGCGGCCGCTCAATGATGATGATGATGATGAGGTCCACCAGGAGTGGTAG | *Not*I |
| *axe1dC* R2 | TCCCCGCGGAGGTCCACCAGGAGTGGTAG | *Sac*II |
| *cbm4-2* R3 | ATAAGAATGCGGCCGCTCAATGATGATGATGATGATGGTCAACGATAGCC | *Not*I |
| *cbm* *6* R4 | ATAAGAATGCGGCCGCTCAATGATGATGATGATGATGATTGACTCCGTTAGAAT | *Not*I |
| *cbm* *22-2* R5 | ATAAGAATGCGGCCGCTCAATGATGATGATGATGATGACCCTCGATAACGG | *Not*I |

^a^ Restriction sites are underlined in the sequence.
