# Supplementary material for: Replacement of carbohydrate binding modules improves acetyl xylan esterase activity and its synergistic hydrolysis of different substrates with xylanase
Source: BMC Biotechnol. 2016 Oct 22;16:73. doi: 10.1186/s12896-016-0305-6 (PMC5075172; doi:10.1186/s12896-016-0305-6)
Supplement: Additional file 3: Figure S1. — Construction of recombinant plasmids. (DOC 57 kb) [file 12896_2016_305_MOESM3_ESM.doc]

(1)

*Eco*RI


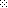


**CD**

F1

R1

*Not*I

**CBM1**

**AXE1**

PCR, digestion and insertion into pPICZαA between *Eco*RI and *Not*I sites

*Not*I

*Eco*RI


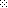


**CD**

**AXE1dC**

(2)

F3

R3

**CBM4-2**

F4

R4

*Eco*RI

F2

R2

*Not*I

**CBM6**


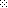


**CD**

**CBM1**

**CBM22-2**

F5

R5

PCR

PCR

*Not*I

*Sac*Ⅱ

**CBM4-2**

*Sac*Ⅱ

*Eco*RI

*Not*I

*Sac*Ⅱ


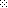


**CD**

**CBM6**

*Not*I

*Sac*Ⅱ

**CBM22-2**

Ligation and insertion into pPICZαA between *Eco*RI site and *Not*I site


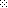


**CD**

*Eco*RI

*Not*I

**AXE1dC-CBM4-2**

*Sac*Ⅱ

**CBM4-2**


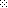


**CD**

*Eco*RI

*Not*I

*Sac*Ⅱ

**CBM6**

**AXE1dC-CBM6**


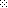


**CD**

*Eco*RI

*Not*I

*Sac*Ⅱ

**CBM22-2**

**AXE1dC-CBM22-2**

Figure S1 Construction of pPICZαA-AXE1dC, pPICZαA-AXE1, pPICZαA-AXE1dC-CBM4-2, CZαA-AXE1dC-CBM6 and pPICZαA-AXE1dC-CBM22-2.
